# Supplementary material for: Diagnostic accuracy of a sequence-specific Mtb-DNA hybridization assay in urine: a case-control study including subclinical TB cases
Source: Microbiol Spectr. 2024 May 8;12(6):e00426-24. doi: 10.1128/spectrum.00426-24 (PMC11237410; doi:10.1128/spectrum.00426-24)
Supplement: Supplemental material — Fig. S1. [file spectrum.00426-24-s0001.docx]

**Supplemental Material**

***DNA degradation tests in urine samples****. (A) Effects of long bench-time and preservation were tested in triplicates. Urines samples were spiked with 10^5^ copies of SPC, left at RT for 24 hours before storage in the presence or not of EDTA + Tris-HCL at -80°C for 1 or 83 days. (B) The effect of cryopreservation were tested individually in SPC-spiked urines samples. Triplicates of 10mL urine samples with 10^5^ copies were stored, in the presence or not of EDTA + Tris-HCL for 24 hours at RT or at -80°C. Two urine triplicates, in the presence or not of EDTA + Tris-HCL were immediately processed as a control for maximal recovery (100%).*

**(A)**

**(B)**
